# P-816. Are We Selecting the Right Patients for FilmArray Blood Culture Testing? Insights from a Gram-Negative Bacteremia Cohort

**DOI:** 10.1093/ofid/ofaf695.1024

**Published:** 2026-01-11

**Authors:** Maximiliano Gabriel Castro, Melina Tatiana Beloso, Ana Paula Amato, Joaquín Ignacio Coduri Anthonioz Blanc, Erwin Alexander Rottoli, Guillermina Cravero, Agustina Agüero, Agustín Martínez, Luciano Priotti, Mateo Pujato, Joaquín Portillo, Gisela Muñoz Cena, Andrea Gómez Colussi, Fernanda Argarañá, Federico Rafael Galluccio

**Affiliations:** CEMIC: Centro de Educacion Medica e Investigaciones Clinicas Norberto Quirno, Ciudad autónoma de Buenos Aires, Ciudad Autonoma de Buenos Aires, Argentina; Hospital Dr. JB Iturraspe, Santa Fe, Santa Fe, Argentina; Hospital Dr. JB Iturraspe, Santa Fe, Santa Fe, Argentina; Hospital Dr. JB Iturraspe, Santa Fe, Santa Fe, Argentina; Hospital Dr. JB Iturraspe, Santa Fe, Santa Fe, Argentina; Hospital Dr. JB Iturraspe, Santa Fe, Santa Fe, Argentina; Hospital Dr. JB Iturraspe, Santa Fe, Santa Fe, Argentina; Hospital Dr. JB Iturraspe, Santa Fe, Santa Fe, Argentina; Hospital Dr. JB Iturraspe, Santa Fe, Santa Fe, Argentina; Hospital Dr. JB Iturraspe, Santa Fe, Santa Fe, Argentina; Hospital Dr. JB Iturraspe, Santa Fe, Santa Fe, Argentina; Hospital Dr. JB Iturraspe, Santa Fe, Santa Fe, Argentina; Hospital Dr. JB Iturraspe, Santa Fe, Santa Fe, Argentina; Hospital Dr. JB Iturraspe, Santa Fe, Santa Fe, Argentina; Hospital Dr. JB Iturraspe, Santa Fe, Santa Fe, Argentina

## Abstract

**Background:**

Gram-negative bacteremia (GNB) is associated with high mortality. The implementation of FilmArray blood culture panels (FAP) has been shown to reduce mortality in patients with sepsis. However, the high cost of these panels limits their routine use in all GNB cases. This study aimed to identify variables associated with opportunities for antibiotic optimization (OAO) using the FAP in GNB, and to evaluate the appropriateness of its current utilization.

**Figure:**
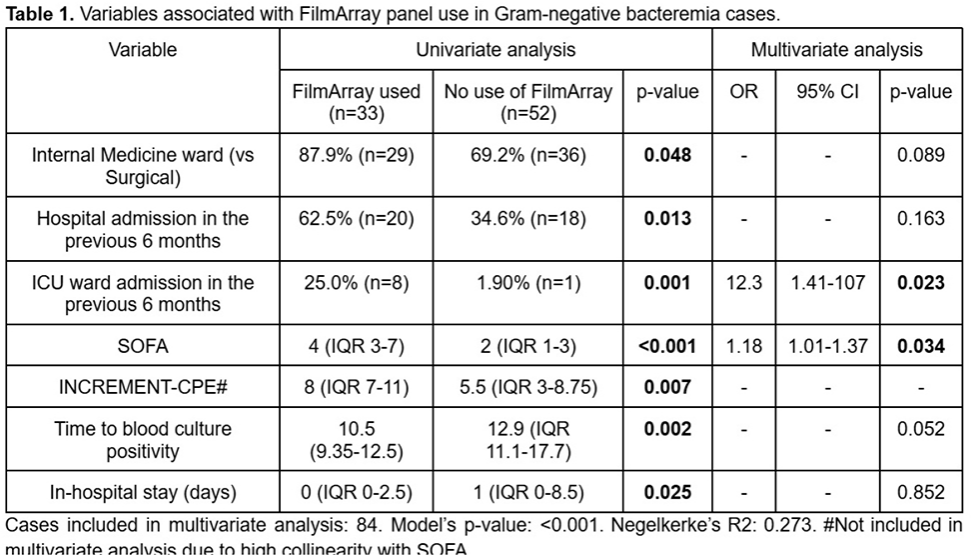

**Figure:**
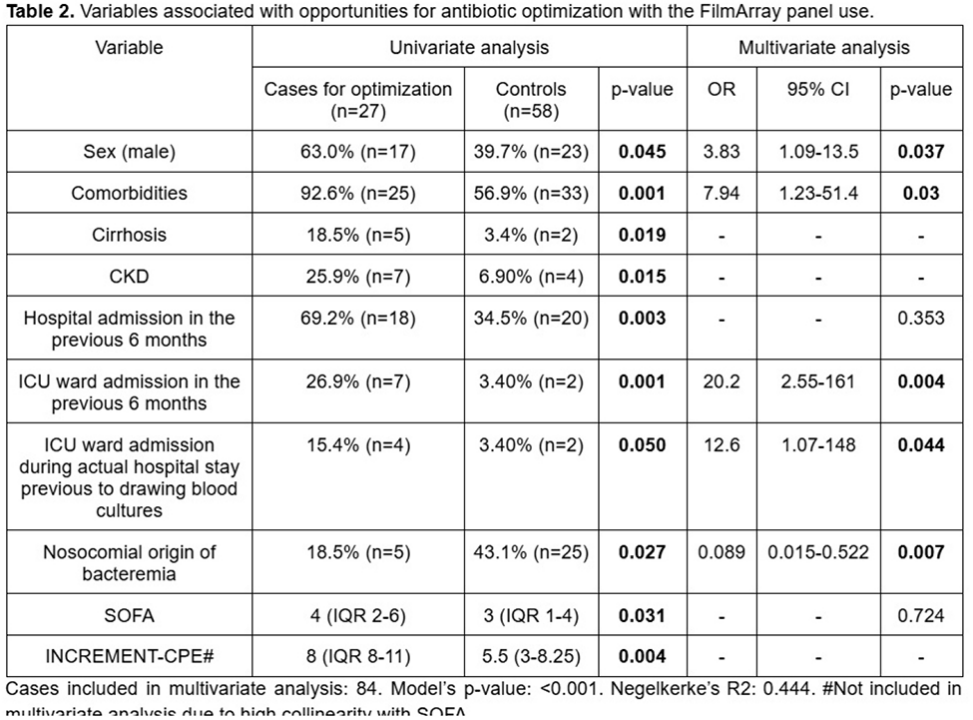

**Methods:**

Ambispective cohort study of patients with GNB hospitalized in general wards at an Argentine hospital. Blood samples were collected using BactAlert® blood culture bottles. Microorganism identification and antimicrobial susceptibility testing were performed using the VITEK® 2C automated system. The FilmArray® BioFire® BCID2 panel was applied selectively, based on clinical judgment by the treating physicians. Opportunities for antibiotic optimization (OAO) using the FAP were defined as the detection of microorganisms or resistance genes likely to require modification of empirical therapy. OAO criteria included the presence of carbapenemase genes, special pathogens (such as *Listeria spp* or *Salmonella spp*.) and, in community-acquired infections, *Pseudomonas aeruginosa* and CTX-M-type extended-spectrum beta-lactamase (ESBL) genes. For statistical analysis, Chi2 and Mann–Whitney U tests were used, and variables with p< 0.05 were included in multivariate logistic regression models.

**Results:**

85 patients were included. FAP was used in 38.8% (n=33) of cases. Variables associated with FAP use are presented in Table 1. OAO were identified in 31.8% (n=27) of the cohort, and were associated with higher mortality (40.7% vs 17.2%, p=0.019). Only 59.3% of OAO cases were tested using the FAP. Variables associated with OAO are shown in Table 2.

**Conclusion:**

In this cohort, FAP use was more frequent in severely ill patients, as indicated by higher SOFA scores and prior Intensive Care unit (UCI) admission. However, broader application may be warranted due to GNB high baseline mortality. Variables significantly associated with OAO—including male sex, presence of comorbidities, community-onset infections, ICU stay during the current hospitalization and prior hospital admissions—did not influence the selection of patients for FAP use.

**Disclosures:**

All Authors: No reported disclosures

